# P374: Enhanced hospital hygienic monitoring program 2011-2012 in a acute regional hospital in Hong Kong

**DOI:** 10.1186/2047-2994-2-S1-P374

**Published:** 2013-06-20

**Authors:** WM Lee

**Affiliations:** 1 Infection Control Team, Princess Margaret Hospital, Hong Kong, Hong Kong; 2 Supporting Services Section (Central Ward Supporting Service), Princess Margaret Hospital, Hong Kong, Hong Kong; 3 International Supporting Service (ISS), Princess Margaret Hospital, Hong Kong, Hong Kong

## Introduction

There is increasing evidence to show that contaminated environment does play a role in transmission of healthcare associated infection. Improvement in the effectiveness of daily and terminal cleansing of high touch surfaces is identified as one of the crucial element in hospital infection prevention. In view of the growing challenge of infections caused by multi-drugs resistant organisms, an infection prevention program for cleansing staff was initiated by infection control team (ICT) in Princess Margaret Hospital.

## Objectives

The program was aim at developing structured education package and standard for cleansing discharge bed and high touch areas, and to develop tools and method for cleansing service provider for performance service monitoring.

## Methods

The program consisted of series education sessions on “before-after practical assessment of cleaning discharge bed” with audio-visual materials. Checklist consists of 10 selected high touch areas around patient bed unit was designed for training and assessment. “Fluorescent marker” was the indicator of cleansing effectiveness.

Cleansing service supervisors conducted monthly assessment on cleansing staff with the checklist provided. ICT conducted audit every 4 - 6 months.

## Results

There was significant improvement (>80%) in staff achievement immediate after training. However, compliance dropped and fluctuated with passage of time as shown in ICN audit in June and Oct 2011 and Jun in 2012. Cleansing effectiveness was below satisfactory level in certain high touch areas.

60 – 70% of both ISS and supporting service staff achieved above 75% recommended score. Median score of both groups was 80 - 90%. Wide range (19 – 100%) was found in ISS. Around 15% ISS staff kept below 50% achievement score in 3 times of audit.

## Conclusion

The training needs of cleansing staff and the importance of continuous performance monitoring were identified. Cleansing work standard was formulated for cleansing a discharge bedand targeted high touch areas. To up keep standard, continuous self-assessment and monitoring by service providers was recommended.

## Disclosure of interest

None declared

